# Out of Sight, Out of Mind or Just Something in the Way? Visual Barriers Do Not Reduce Intraspecific Agonism in an All-Male Group of Nile Crocodiles (*Crocodylus niloticus*)

**DOI:** 10.3390/ani12030269

**Published:** 2022-01-22

**Authors:** Austin Leeds, Alex Riley, Megan Terry, Marcus Mazorra, Lindsay Wick, Scott Krug, Kristen Wolfe, Ike Leonard, Andy Daneault, Andrew C. Alba, Angela Miller, Joseph Soltis

**Affiliations:** 1 Animals, Science and Environment, Disney’s Animal Kingdom^®^, Lake Buena Vista, FL 32830, USA; Alex.Riley@disney.com (A.R.); Megan.E.Terry@disney.com (M.T.); Marcus.E.Mazorra@disney.com (M.M.); Lindsay.E.Wick@disney.com (L.W.); Scott.W.Krug@disney.com (S.K.); Kristen.Wolfe@disney.com (K.W.); Ike.A.Leonard@disney.com (I.L.); Andre.J.Daneault@disney.com (A.D.); Andrew.Alba@disney.com (A.C.A.); Angela.Miller@disney.com (A.M.); Joseph.Soltis@disney.com (J.S.); 2 New College of Florida, Sarasota, FL 34243, USA

**Keywords:** animal behavior, animal welfare, zoo exhibit design, visual barrier, reptile behavior, reptile welfare, crocodilian, evidence-based management

## Abstract

**Simple Summary:**

The behavior of animals living in zoos and aquariums is influenced by the exhibits they live in, similar to how the behavior of animals in nature is influenced by the ecosystem they inhabit. In zoos and aquariums, changes in exhibit design can be implemented to modify the behavior of animals to ensure they are experiencing optimal welfare. Here, we evaluated if the addition of visual barriers—physical barriers placed at the surface of the water—reduce aggression amongst male Nile crocodiles living in a zoo. Both short- and long-term monitoring found that visual barriers did not reduce aggression within the group. While ineffective at reducing aggression, this study represents the first evaluation of exhibit design in relation to the behavior of a crocodilian species in a zoo or aquarium setting. As a commonly managed taxa in zoos and aquariums, it is imperative that their behavior and welfare are assessed systematically. We hope the methodologies and learnings from this study encourage future study of crocodilian behavior and welfare.

**Abstract:**

Here, we evaluated if visual barriers could reduce intraspecific agonism in an all-male group of Nile crocodiles (*Crocodylus niloticus*) living in a zoo. Crocodiles were monitored for nearly 100 h, and four “hotspots” of aggression within their exhibit were identified. Within these four locations, visual barriers were placed at the surface of the water with the goal of reducing agonism by targeting sight lines associated with their species-typical minimum exposure posture, where crocodiles submerge their body but maintain facial sensory organs above the water line. Crocodile behavior was then monitored for 226 h, evaluating both short- and long-term effects of the visual barriers. In both observation periods, intraspecific agonism was unaffected by visual barriers. However, crocodiles were more likely to be on land and closer together, after the barriers were installed, showing the barriers affected nonagonistic behaviors. Monitoring of such unintended effects is significant to ensure no welfare concerns are created in any exhibit or husbandry modification attempt. Additionally, time of day and temperature were significant predictors of behavior, highlighting the importance of such factors in the analysis of reptilian behavior. While ineffective at reducing agonism, this is the first published study evaluating exhibit design and behavior of crocodilians in zoos and aquariums. The methodologies and findings here should provide useful information for future behavioral and welfare studies of this understudied taxa.

## 1. Introduction

The behavior of animals in zoos and aquariums is significantly influenced by the exhibits they inhabit. For example, animal behavior has been shown to vary in relation to exhibit complexity [1,2], indoor and outdoor space availability [3,4], and how time is managed between multiple exhibit and holding spaces [5,6,7]. The addition of visual barriers—structural elements implemented in or around exhibits to limit visual access of animals to specific stimuli through hiding, privacy, and/or camouflage [8,9]—is another exhibit element that has broad applicability to influence animal behavior.

The main function of visual barriers is to disrupt sight lines between an animal and stimuli associated with the behavior of interest. In most contexts, the goal is to reduce a specific undesirable behavior; thus, the visual barrier attempts to create an “out of sight, out of mind” situation for the animal in regard to the target stimuli. For example, inclusion of visual barriers has been associated with decreased rates of intraspecific agonism in domestic pigs (*Sus domesticus*, [10]), pheasants (*Phasianus colchicus*, [11]), tilapia (*Tilapia rendalli*, [12]), and marabou storks (*Leptoptilos crumeniferus*, [13]) by disrupting sight lines between conspecifics. Similarly, visual barriers disrupting sight lines between adjoining habitats of okapi (*Okapia johnstoni*), a solitary species, was associated with a decrease in stereotypic behavior [14], and the placement of a visual barrier between a capuchin monkey (*Cebus apella*) and a neighboring carnivore enclosure reduced alarm calls and vigilance behavior in the monkeys [15]. Reviewing 20 years of breeding records from two in situ conservation centers, Flanagan et al. [16] found that physical distance between pairs of ‘Alalā (*Corvus hawaiiensis*) was significantly associated with reproductive success; however, the placement of visual barriers between adjoining aviaries was an effective proxy for replicating the effects of physical distance when increasing physical distance between pairs was not possible. The relationship between animal behavior and visitor presence is variable [17]; however, placement of visual barriers that distort visitor presence has been associated with positive changes in behavior including decreased vigilance in little penguins (*Eudyptula minor*, [18]) and decreased intraspecific agonism, stereotypic behavior, and vigilance in gorillas (*Gorilla gorilla gorilla*, [19,20]).

However, visual barriers are not always effective. Kuhar et al. [21] found that tamarins (*Saguinus geoffroyi*) housed adjacent to conspecific groups, but separated by visual barriers, had higher rates of agonism than tamarin groups not housed adjacent to conspecific groups, suggesting the visual barriers were ineffective at minimizing behavior associated with the presence of neighbors. Bashaw et al. [22] found that pacing in a Sumatran tiger (*Panthera tigris sumatrae*) increased following the placement of a visual barrier between a neighboring conspecific enclosure and animal caregiver workspace, both of which were thought to be stimuli associated with the occurrence of pacing in baseline conditions. Additionally, a visual barrier placed on a visitor viewing window had no effect on the visitor-directed agonistic behavior and stereotypic behavior of a male drill (*Mandrillus leucophaeus poensis*) who was known to be sensitive to visitor presence [23].

Though agonism is a species-typical behavior and an aspect of maintaining social relationships and groupings for many species (e.g., [24]), excessive agonism, particularly agonism that results in physical injury, can be significantly detrimental to the welfare of animals in zoos and aquariums [25,26,27,28,29]. Thus, managers of animals in zoos and aquariums have a significant responsibility to provide settings that do not result in atypical levels of agonism. Reducing agonism can be challenging, but exhibit modifications, such as those including the previously described visual barriers, are often an easily implemented first step. To date, only one study has evaluated the effect of visual barriers on the agonistic behavior of a reptile. Brien et al. [30] tested the effect of visual barriers on the agonistic behavior of hatchling saltwater crocodiles (*Crocodylus porosus*). In experimental tanks, a visual barrier in the form of a suspended plastic grid covering a large proportion of the available water surface was installed. This visual barrier was designed to replicate the natural visual barriers created by floating vegetation at the edges of aquatic environments inhabited by hatchling crocodiles in nature. Crocodiles in tanks with visual barriers had lower rates of agonism compared with crocodiles in tanks with no visual barriers. These data preliminarily suggest that visual barriers placed at the surface of the water may be an effective strategy for reducing agonism in crocodiles living in human care. However, the findings reported by Brien et al. [30] may have limited applicability to the care and welfare of crocodiles in zoos and aquariums as only hatchling behavior was evaluated and the visual barrier design presents scalability challenges in larger exhibits.

Regardless, the results of Brien et al. [30] provide a starting point for evaluating the effectiveness of visual barriers to mitigate crocodilian intraspecific agonism in zoos and aquariums. Particularly, their approach targeted an important crocodilian behavior—the minimum exposure posture. This posture allows individuals to keep sensory anatomy (eyes, nostrils, ears, cranial platform) exposed at the water line while the rest of their body remains concealed below the surface [31]. Important in ambush hunting of prey, this posture also helps visually detect conspecifics and is associated with the visual signals utilized in social interactions at the surface of the water [31,32]. Visual barriers aimed at disrupting sight lines associated with this minimum exposure posture may be beneficial for reducing agonism. While the Brien et al. [30] study highlighted that the design of their visual barriers was reflective of the natural environment of hatchling crocodiles, its design also targeted the minimum exposure posture by placing visual barriers right at the surface of the water. For those managing adult crocodilians in larger habitats (e.g., zoos and aquariums), designing visual barriers that target the minimum exposure posture may be effective for minimizing intraspecific agonism.

Nile crocodiles (*Crocodylus niloticus*) are large, apex predators who occupy freshwater habitats across Africa [33]. An in-depth understanding of this species’ sociality is lacking; however, data suggest that crocodile population densities can be seasonally dependent [34] and males appear to form territories that they defend from other adult males [35,36]. Unfortunately, data describing long-term association patterns and how crocodiles group and interact from a more day-to-day perspective in nature are not part of the published literature. Disney’s Animal Kingdom^®^ is home to a large, all-male group of Nile crocodiles. Animal care staff have identified the mitigation of intraspecific agonism as an area of focus in the day-to-day care and management for this group. To date, monitoring has shown a seasonal pattern in the crocodiles’ agonistic behavior [Disney’s Animal Kingdom^®^, unpublished data] and that guest presence does not increase intraspecific agonism [37]. The inclusion of visual barriers within their habitat has occurred previously but has not received focused, systematic monitoring to evaluate effectiveness. The purpose of this study is to empirically evaluate the effectiveness of newly installed visual barriers to reduce intraspecific agonism within this group of crocodiles during the winter months when seasonal increases in agonism are observed. This study represents the first evaluation of visual barriers on the social behavior of crocodilians in a zoo, findings from which should be informative to the care and welfare of this commonly managed but poorly studied order.

## 2. Materials and Methods

### 2.1. Study Subjects and Visual Barriers

A group of 21 adult male Nile crocodiles living at Disney’s Animal Kingdom^®^ Theme Park, Lake Buena Vista, Florida, ranging in age from 32 to 38 years, were observed for this study. The crocodiles live in an open air, outdoor habitat containing open water, beaches, and islands (for additional exhibit information see [37]). Water temperature was controlled to maintain consistent temperatures, averaging 77.8 °F (SE = 0.07) during the study periods.

**Figure 1:**
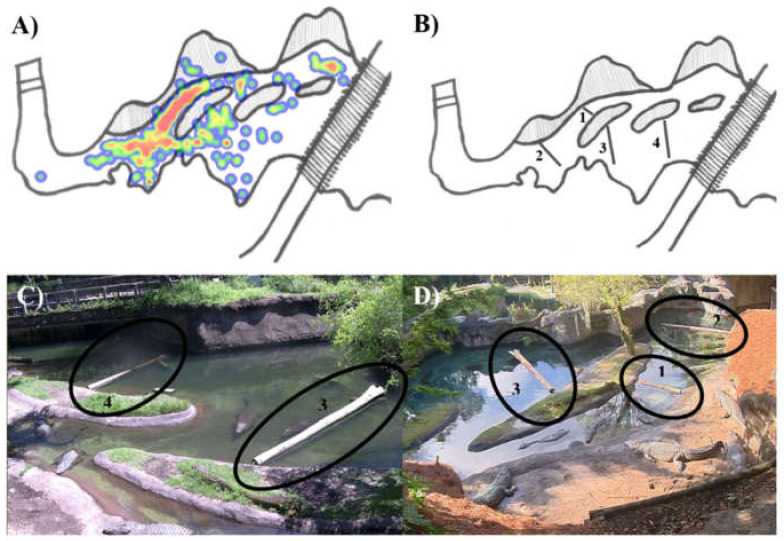
(**A**) Heatmap depicting the locations of agonistic interactions observed in April and May 2020. Locations highlighted in red are more frequent locations of agonism. Locations highlighted in green and dark blue are less-frequent locations of agonism. Heatmap generated using the ZooMonitor application [38]. (**B**) Map of exhibit highlighting placement of visual barriers (labeled 1–4). (**C**,**D**) Images of exhibit with visual barriers highlighted.

Four visual barriers were installed in the exhibit in October 2020. Placement was selected using a data-driven process. Prior to visual barrier installation, crocodile behavior was monitored for seven weeks (97 observation hours) during which locations of agonistic encounters were mapped within the exhibit (Figure 1). From these data, three “hotspots” of agonism were identified, which were primarily in shallow water near the main beach and in the deeper water immediately adjacent to the main beach. Visual barriers were placed in these three locations. An additional visual barrier was placed in a location not identified as a “hotspot” but adjacent to the “hotspot” areas with similar features (Figure 1). Visual barriers were large wooden logs with one end mounted to an island or beach that then extended out into the water. Visual barriers floated but were additionally supported by submerged concrete traffic barriers to minimize waterlogging-related sinking and side-to-side movement. Visual barriers labeled 1, 2, 3, and 4 were approximately 5, 20, 20, and 16 ft in length, respectively.

### 2.2. Data Collection

Data were collected as part of an ongoing, long-term monitoring study of this group’s behavior, methods for which have been previously described [37]. Data were collected during one-hour observations conducted between 7 a.m. and 5 p.m. up to twice daily. Observation times were randomly selected and balanced across all hours. Observations were conducted via coding video recorded from remote cameras that covered >90% of the exhibit, providing greater exhibit visibility than if conducted in person while removing observer presence as a potential confounding variable. Agonistic behavior was recorded using all occurrence sampling, and space use and social proximity were collected via three group scans per observation (0 min, 30 min, and 60 min). Data were collected by authors AL, AR, and ACA, as well as four interns of the Science Operations team at Disney’s Animal Kingdom^®^. Interobserver reliability was established amongst all collectors by double-coding one-hour videos and maintaining at least 80% agreement for each behavioral measure.

Rates of agonistic behavior were recorded and defined as aggressive or intolerant behaviors directed from one crocodile towards a conspecific resulting in physical contact [39], including bites and jaw clashes. A bite was defined as one crocodile closing one’s jaws around a conspecific, possibly including a roll or shake. Jaw clash was defined as two crocodiles striking heads together with their mouths open. Agonistic behavior was recorded at the bout level, where a bout began with the initiation of the behavior and ended after a period of five seconds where no additional behaviors occurred. In addition, space use and social proximity measures were collected. For space use, the number of crocodiles in the water were counted. If a crocodile had part of their body both in water and on land, they were counted as in water if the majority (>50%) of their body was in water. For social proximity, the number of crocodiles in physical contact with a conspecific were counted.

Data were collected to evaluate the effectiveness of the visual barriers in two ways. The first was a short-term effect evaluation. Data were collected for ten days (20 h of observation) from 22 September through 1 October 2020 prior to visual barrier installation (previsual barrier). The crocodiles were then shifted to an off-exhibit holding area on 2 October while barrier installation began. Beginning 8 October and ending 13 October 2020, small subgroups of crocodiles were reintroduced to the habitat. The crocodiles were allowed to reacclimate to the exhibit as a group for three days; subsequently, ten days of data (20 h of observation) were collected to evaluate behavior in the presence of the visual barriers (post-visual-barrier).

A long-term effect evaluation was then conducted. Behavior was observed over two months following the installation of visual barriers in November (44 observation hours) and December 2020 (49 observation hours). Previous monitoring of this group has shown a distinct seasonality in the behavior of the crocodiles [Disney’s Animal Kingdom^®^, unpublished data]. To account for this, a match–control methodology was used for the long-term monitoring, where these post-visual-barrier observations were compared to observations collected during the same months the previous year [37]. Pre-visual barrier observations occurred in November (44 observation hours) and December 2019 (49 observation hours).

### 2.3. Data Analysis

Generalized linear mixed models were used to analyze crocodile behavior. For both short- and long-term analysis, four models were run with total agonistic bouts, agonistic bouts on land, space use, and social proximity as dependent variables. Analysis of agonistic behavior was divided into two measures (total agonism and agonism on land) as preliminary evaluation of the data suggested that agonism may have increased on land following the visual barrier installation, aligning with an initially discussed potential that the visual barriers may simply move agonism to new locations rather than reduce it. The inclusion of on land agonism as a dependent variable was to evaluate this potential occurrence. All models were conducted in R (v 4.0.5, [40]) using the glmmTMB function [41]. For both agonistic bout models, the dependent variable was a count of the number of agonistic interactions observed per observation. Models were run using a zero-inflated Poisson distribution and a log link function as visual inspection of the data showed that count values of 0 were greater than any other value. For space use, the dependent variable was the proportion of the group in water. For social proximity, the dependent variable was the proportion of the group in proximity to a conspecific. Both the space use and proximity models were run using a binomial distribution and a logit link function. All models included observation day as a random variable. Barrier presence (yes/no), time of day (morning, 7 a.m. through 10 a.m.; midday 11 a.m. through 2 p.m.; afternoon, 3 p.m. to 5 p.m.) and temperature (°F at end of observation) were included as fixed factors in all models. In addition, month (November/December) was included as a fixed factor in the long-term analysis models to account for seasonality. Time of day, temperature, and month were included as control factors, as a previous study found that they significantly affect the behavior of this group [37]. Multicollinearity was assessed by variance inflation factor testing using the vif function. All variance inflation factor scores were less than 2, indicating that multicollinearity was not affecting analysis [42]. Regression residuals were visualized using a Q-Q plot to assess normality, with no obvious deviations, suggesting a strong model fit. Significance was set at α ≤ 0.05 for all tests. Post hoc multiple comparison tests used a Tukey method adjustment.

## 3. Results

### 3.1. Short-Term Effects

**Figure 2:**
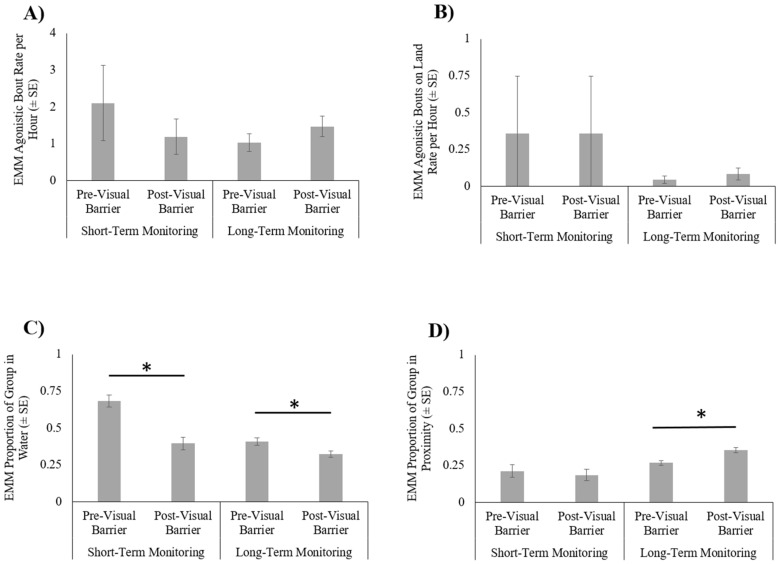
Crocodile behavior (estimated marginal mean (EMM) ± SE) pre- and post-visual-barrier installation during both short- and long-term monitoring periods. (**A**) Rate of agonistic bouts. (**B**) Rate of agonistic bouts on land. (**C**) Proportion of group in water. (**D**) Proportion of group in proximity to conspecific. Asterisks (*) denote statistical significance (*p* ≤ 0.05).

**Figure 3:**
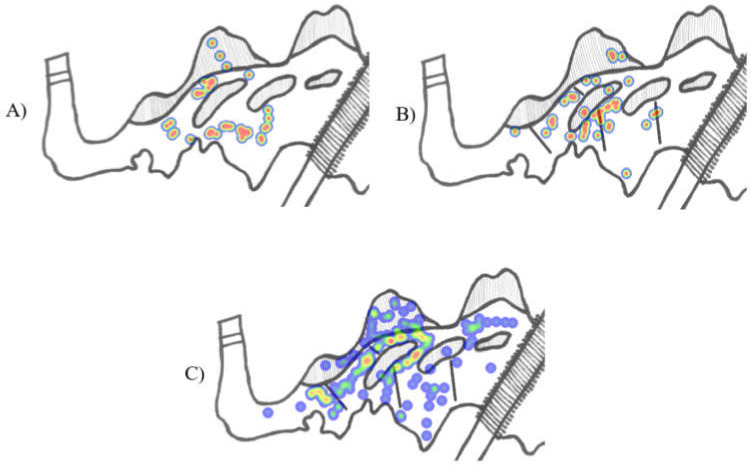
(**A**) Heatmap depicting the locations of agonistic interactions observed during the short-term pre-visual barrier observation period. (**B**) Heatmap depicting the locations of agonistic interactions observed during the short-term post-visual-barrier observation period. (**C**) Heatmap depicting the locations of agonistic interactions observed during the long-term post-visual-barrier observation period. Note: data for long-term pre-visual barrier observation period were not recorded in a visual format. All heatmaps generated using the ZooMonitor application [38]. Locations highlighted in red are more-frequent locations of agonism. Locations highlighted in green and dark blue are less-frequent locations of agonism.

Rate of total agonistic behavior did not differ between pre- (µ = 2.10, SE = 1.019) and post-visual barrier installation (µ = 1.19, SE = 0.479; χ^2^ = 0.514, df = 1, *p* = 0.473; Figure 2; Appendix A). Total agonistic behavior was also not affected by time of day (χ^2^ = 3.228, df = 2, *p* = 0.199) or temperature (χ^2^ = 1.089, df = 1, *p* = 0.297). Rate of agonistic behavior on land did not differ between pre- (µ = 0.358, SE = 0.389) and post-visual barrier installation (µ = 0.461, SE = 0.389; χ^2^ = 0.056, df = 1, *p* = 0.813; Figure 3 for visual of agonistic bout locations). Agonistic behavior on land was also not affected by time of day (χ^2^ = 1.364, df = 2, *p* = 0.506) or temperature (χ^2^ = 1.465, df = 1, *p* = 0.226).

Space use patterns were significantly predicted by the presence of visual barriers (χ^2^ = 21.590, df = 1, *p* < 0.001) with a greater proportion of crocodiles observed in water previsual barrier installation (µ = 0.684, SE = 0.04) than post-installation (µ = 0.396, SE = 0.043; Figure 2; Appendix A). In addition, temperature was positively correlated to the proportion of individuals in water (χ^2^ = 13.585, df = 1, *p* < 0.001) and time of day predicted space use (χ^2^ = 43.543, df = 2, *p* < 0.001). Proportion of group in water decreased throughout the day, with a greater proportion in water in the morning (µ = 0.701, SE = 0.033) than midday (µ = 0.548, SE = 0.037; *p* < 0.001) and afternoon (µ = 0.373, SE = 0.044; *p* < 0.001) and a larger proportion of the group in water midday than afternoon (*p* < 0.001).

Proximity patterns did not differ between pre- (µ = 0.212, SE = 0.043) and post-visual barrier installation (µ = 0.185, SE = 0.038; χ^2^ = 0.217, df = 1, *p* = 0.299; Figure 2; Appendix A). Temperature was negatively associated with the proportion of individuals in proximity (χ^2^ = 4.468, df = 2, *p* = 0.035). Proportion of individuals in proximity also significantly varied by time of day (χ^2^ = 42.525, df = 2, *p* < 0.001). The proportion of crocodiles in proximity was greatest at midday (µ = 0.321, SE = 0.043) and significantly lower in the morning (µ = 0.154, SE = 0.028; *p* < 0.001) and afternoon (µ = 0.150, SE = 0.030; *p* < 0.001).

### 3.2. Long-Term Effects

Rate of total agonistic bouts did not significantly differ between pre- (µ = 1.03, SE = 0.243) and post-visual barrier installation observations (µ = 1.47, SE = 0.282; χ^2^ = 2.433, df = 1, *p* = 0.119; Figure 2; Appendix B). Temperature (χ^2^ = 0.114, df = 1, *p* = 0.736) and month (χ^2^ = 2.672, df = 1, *p* = 0.102) similarly were not a significant predictor of total agonistic bouts. Time of day significantly predicted total agonistic bouts (χ^2^ = 26.010, df = 2, *p* < 0.001), with agonism occurring more frequently in the morning (µ = 2.364, SE = 0.377) than midday (µ = 0.646, SE = 0.153; *p* < 0.001). Though not statistically significant, average morning rates were greater than afternoon rates (µ = 1.216, SE = 0.474; *p* = 0.250) and afternoon rates were greater than midday rates (*p* = 0.263). Rate of agonistic bouts on land did not significantly differ between pre- (µ = 0.044, SE = 0.026) and post-visual barrier installation observations (µ = 0.084, SE = 0.042; χ^2^ = 1.649, df = 1, *p* = 0.199; Figure 3 for visual of agonistic bout locations). Time of day (χ^2^ = 2.673, df = 2, *p* = 0.263), temperature (χ^2^ = 0.244, df = 1, *p* = 0.622), and month (χ^2^ = 1.034, df = 1, *p* = 0.309) were also not significant predictors of on land agonism.

Space use patterns of the group were significantly predicted by the presence of visual barriers (χ^2^ = 7.251, df = 1, *p* = 0.007) with a greater proportion of the group utilizing the water pre-visual barrier installation (µ = 0.408, SE = 0.025) than post-installation (µ = 0.323, SE = 0.021; Figure 2; Appendix B). Water use was also negatively associated with temperature (χ^2^ = 33.278, df = 1, *p* < 0.001) and varied by time of day (χ^2^ = 166.543, df = 2, *p* < 0.001). A greater proportion of crocodiles spent time in water in the morning (µ = 0.540, SE = 0.02) than midday (µ = 0.257, SE = 0.017; *p* < 0.001) and afternoon (µ = 0.317, SE = 0.025; *p* < 0.001) and more spent time in water in the afternoon than midday (*p* = 0.008). Month did not significantly predict space use (χ^2^ = 0.059, df = 1, *p* = 0.809).

Proximity patterns were significantly predicted by the presence of visual barriers (χ^2^ = 13.707, df = 1, *p* < 0.001), with the proportion of individuals in proximity to conspecifics being greater post-visual-barrier installation (µ = 0.356, SE = 0.018) than pre-installation (µ = 0.268, SE = 0.017; Figure 2; Appendix B). In addition, time of day also significantly predicted proximity of group members (χ^2^ = 57.583, df = 2, *p* < 0.001). A greater proportion of the group was in proximity in the afternoon (µ = 0.385, SE = 0.023) than morning (µ = 0.220, SE = 0.012; *p* < 0.001) and greater at midday (µ = 0.340, SE = 0.017) than in the morning (*p* < 0.001). Afternoon proportions approached significance to being greater than midday proportions (*p* = 0.059). Temperature (χ^2^ = 1.929, df = 1, *p* = 0.165) and month (χ^2^ = 1.063, df = 1, *p* = 0.303) did not significantly predict proximity patterns.

## 4. Discussion

Visual barriers were installed in a Nile crocodile habitat with the goal of mitigating intraspecific agonism. Specifically, the visual barriers were intended to disrupt conspecific sight lines associated with the species-specific minimum exposure posture. Both short- and long-term monitoring found that these visual barriers were ineffective at reducing intraspecific agonism. Here, visual barriers were installed in locations where agonistic interactions frequently occurred, supported by nearly 100 h of observation, minimizing the chance that visual barriers were ineffective at reducing agonism simply because their locations within the exhibit were insufficient. A previous study has shown that similarly functional visual barriers almost eliminated agonism within groups of hatchling saltwater crocodiles [30], suggesting that targeting the minimum exposure posture has value in crocodilian agonism mitigation.

The design and installation of the visual barriers may have been a contributing factor to the results observed here. First, while the visual barriers were used to disrupt sight lines between conspecifics, crocodiles can also perceive the presence of conspecifics through vibrational, chemical [43], and auditory means [32]. These communication or sensory mediums were not monitored here; however, it is likely that these signals were unaffected by the visual barriers, resulting in the crocodiles still being aware of the physical presence of conspecifics nearby despite disrupted sight lines. Second, the density of the visual barriers within the habitat was low. Brien et al. [30] found that creating a grid-style visual barrier evenly distributed across the entirety of the available water surface was effective at reducing agonism in hatchling crocodiles. While covering the entirety of a habitat may not be necessary to reduce agonism, there may be a minimum amount of visual barrier relative to total water surface area that is effective at reducing agonism. Here, it was estimated that visual barriers covered approximately 1% of the crocodiles’ water surface area; thus, while the visual barriers were systematically placed, they may have been insufficient in regard to their ability to displace sight lines within a large exhibit and amongst a large number of crocodiles.

Given these two significant limitations, it is possible the barriers utilized here did not function as a true visual barrier but were rather just an added physical barrier between conspecifics, easily circumvented by submerging several inches below the water surface. Future evaluations of visual barriers with crocodilians may benefit from the inclusion of barriers below the water line to increase the challenge of directly reaching conspecifics as well as disrupting sight lines below the water surface. When considering optimal designs for zoo and aquarium settings, Brien et al. [30] should be the starting point, as their methods were effective at reducing intraspecific agonism. While replicating their exact approach is challenging in a zoo or aquarium setting, the principles of increasing exhibit complexity and implementing whole exhibit interventions are achievable. For example, dying exhibit water to increase opacity may disrupt visual access between conspecifics and adding aquatic plants may create additional water level and submerged visual barriers that can cover a large proportion of an exhibit. Though agonism was not reduced, it is worth highlighting that it also did not increase. A potential side effect of visual barriers, in any context, is they may create “dead ends” within a habitat where individuals could be trapped by more dominant animals. The lack of this occurrence here, as assessed by the flat rate of agonism and space use data indicating that rates of agonism did not increase around the barriers (Figure 3), is encouraging for future crocodilian visual barrier evaluations.

The success of minimizing intraspecific agonism is also likely dependent on understanding the underlying motivations of the agonistic interactions themselves. The present study occurred during the winter, which has been associated with increased agonism in this group [Disney’s Animal Kingdom^®^, unpublished data]. It is hypothesized that this change in behavior is associated with breeding season physiological changes, though this has not been explicitly tested in this group. Data from sub-Saharan Africa report the breeding season to occur during the cooler months of the year in June and July [44,45]. In this group, behavior changes peak November through January. Despite being temporally different from the sub-Saharan Africa breeding season, both time periods are the coolest months of the year, suggesting that behavior change is associated with the winter breeding season. The visual barriers implemented in this study may not have been a significant enough deterrent to disrupt seasonal changes in behavior linked to their reproductive cycle that encourage displacement of potential competitor males from their immediate space [35,44]. When exhibits are altered in an attempt to reduce an undesirable behavior, the underlying cause(s) of the behavior should be considered in the design and implementation of the alteration.

In addition to the main research question evaluating the visual barriers’ effects on intraspecific agonism, we also evaluated unintended changes in space use and social proximity in response to the visual barriers. In both the short- and long-term monitoring, we observed a significant change in space use, with a greater proportion of the group in the water prior to their installation. The magnitude of change in the short-term monitoring was noticeably larger than the relatively small change observed in the long-term monitoring. This suggests that the short-term response may have been an initial neophobic reaction by the crocodiles to these novel items in their enclosure that decreased over time. In the long-term monitoring, the overall difference on average equated to approximately two less crocodiles in the water, suggesting an overall small group level response for the twenty-one total crocodiles. A potential side effect of the visual barriers was that they may displace agonism to new locations rather than preventing it. Despite the increase in number of crocodiles on land, no change in agonism occurrences on land were observed, suggesting that the visual barriers did not relocate agonism to different locations, which is an encouraging finding for future evaluations. A second potential side effect of the barriers was that they may disrupt how the crocodiles utilized their space, particularly in their locomotor routes and preferred “home ranges”. While we did not specifically monitor these two aspects of their space use, the lack of increased agonism suggests that locomotor routes and “home ranges” were not affected to the degree that individuals who would typically seek to avoid each other were now placed in more frequent contact with one another. Even though the data suggest the long-term effects are minor, it is significant that they were monitored here. Crocodiles are ectotherms and rely on their external environment to regulate their physiology [46,47]. Behavior changes, particularly those involving space use, can thus have significant effects on thermoregulation, ultimately affecting their health and welfare [48]. Ensuring that the ability to thermoregulate is unaffected should be included in any evaluation of ectotherm exhibit modifications, particularly for individuals living in outdoor exhibits where environmental conditions vary.

Social proximity was unaffected in the short-term; however, in the long-term, there was an increase in the proportion of individuals in physical contact following the installation of visual barriers. Estep and Baker [49] found that visual barriers reduced the proximity of stump-tailed macaques (*Macaca arctoides*), likely contributing to their observed decrease in intraspecific agonism. Our data suggest that the visual barriers had the opposite effect, as approximately two more crocodiles, on average, were in contact with conspecifics post-visual barrier compared with pre-conditions. For a group of twenty-one individuals, this statistically significant finding may not be biologically significant. Our finding that rates of agonism on land did not change between conditions, despite the increase in proximity, supports this interpretation as the proximity changes were mostly associated with increased land use.

Finally, we again found that time of day and temperature continue to be significant predictors of crocodilian behavior [37]. We hope that the work presented here inspires future applied work in regard to the husbandry and welfare of crocodilians in human care. In doing so, we emphasize the importance in accounting for these environmental variables when interpreting the success of efforts. Our initial internal sharing of these results, where we use general descriptive statistics to communicate findings of ongoing monitoring between relevant stakeholders, found these visual barriers to be effective at reducing agonism during the short-term evaluation. It was not until we placed our data into our models that we found these differences were not statistically significant, and in fact, become visually reduced when accounting for environmental variables. This is particularly important for institutions who may be doing such evaluations in the absence of staff familiar with advanced modeling statistics. In such cases, conducting observations at the same time of day may be the best option to control for various external factors or comparing observations within set time blocks if round-the-clock monitoring is needed. Additionally, these results highlight the importance of long-term monitoring, as findings here differed by length of study. For example, temperature was seen to have opposite effects on space use between conditions. This may be reflective of a broader temperature range observed in the long-term monitoring compared with the short-term monitoring creating different behavioral patterns. We encourage institutions evaluating the behavior and welfare of any species to consider long-term effects in their evaluation efforts.

## 5. Conclusions

Evaluations of exhibit design on the behavior of animals in zoos and aquariums has significantly contributed to individual species’ care and welfare [1,2,3,4,5,6,7]. Despite over 180 zoo and aquarium facilities managing crocodilian taxa in North America [50], this is the first published study evaluating the relationship between exhibit design and crocodilian behavior. Visual barriers implemented in this study were not effective at reducing intraspecific agonism within a large, all-male group of Nile crocodiles; however, there remains significant opportunity and potential for future evaluations of exhibit design on crocodilian behavior both with this group and within the larger zoo and aquarium crocodilian population. We hope the methodology and findings reported here encourage more detailed evaluations of this understudied taxa’s behavior and welfare. Particularly, multi-institution evaluations of crocodilian behavior should be highly informative in understanding both general behavior and specific behavioral responses (e.g., changes in response to exhibit modification), moving this taxa’s study perspective from case studies to true population patterns.

## Data Availability

Requests for data should be sent to the corresponding author and the scientific review committee of Disney’s Animal Kingdom^®^ for consideration.

## Appendix A. Short-Term Effect Model Outputs

**Table A1:** Statistical outputs for models of the short-term monitoring period.

		Model Estimate Parameters				Analysis of Variance Type III Parameters		
Dependent Variable	Fixed Effects	Estimate	S.E.	Z	*p*	χ^2^	Df	*p*
Rate of Total Agonistic Bouts	Intercept	7.261	6.135	1.184	0.237	1.401	1	0.237
	Barrier Presence (Pre)	0.371	0.517	0.717	0.473	0.514	1	0.473
	Time of Day (Morning)	−1.186	0.917	−1.294	0.196	3.228	2	0.199
	Time of Day (Midday)	−1.219	0.680	−1.792	0.073	-	-	-
	Temperature	−0.075	0.072	−1.043	0.297	1.089	1	0.297
Rate of Agonistic Bouts on Land	Intercept	14.698	12.451	1.180	0.238	1.393	1	0.238
	Barrier Presence (Pre)	−0.253	1.069	−0.237	0.813	0.056	1	0.813
	Time of Day (Morning)	−1.790	2.170	−0.825	0.410	1.364	2	0.506
	Time of Day (Midday)	−2.112	1.808	−1.168	0.243	-	-	-
	Temperature	−0.175	0.145	−1.210	0.226	1.465	1	0.226
Proportion of Group in Water	Intercept	−6.604	1.518	−4.351	<0.001 *	18.932	1	<0.001 *
	Barrier Presence (Pre)	1.195	0.257	4.647	<0.001 *	21.590	1	<0.001 *
	Time of Day (Morning)	1.374	0.208	6.598	<0.001 *	43.543	2	<0.001 *
	Time of Day (Midday)	0.713	0.168	4.240	<0.001 *	-	-	-
	Temperature	0.068	0.018	3.723	<0.001 *	13.858	1	<0.001 *
Proportion of Group in Proximity	Intercept	1.831	1.764	1.038	0.300	1.077	1	0.299
	Barrier Presence (Pre)	0.168	0.360	0.466	0.641	0.217	1	0.641
	Time of Day (Morning)	0.024	0.237	0.100	0.921	42.525	2	<0.001 *
	Time of Day (Midday)	0.980	0.182	5.393	<0.001 *	-	-	-
	Temperature	−0.045	0.021	−2.114	0.035 *	4.468	1	0.035 *

* Denotes statistical significance (α ≤ 0.05).

## Appendix B. Long-Term Effect Model Outputs

**Table A2:** Statistical outputs for models of the long-term monitoring period.

		Model Estimate Parameters				Analysis of Variance Type III Parameters		
Dependent Variable	Fixed Effects	Estimate	S.E.	Z	*p*	χ^2^	Df	*p*
Rate of Total Agonistic Bouts	Intercept	0.851	0.966	0.880	0.379	0.775	1	0.379
	Barrier Presence (Pre)	−0.358	0.300	−1.560	0.119	2.433	1	0.119
	Time of Day (Morning)	0.664	0.416	1.597	0.110	26.010	2	<0.001 *
	Time of Day (Midday)	−0.633	0.404	−1.565	0.117	-	-	-
	Temperature	−0.004	0.013	−0.337	0.736	0.114	1	0.736
	Month (November)	−0.391	0.239	−1.635	0.102	2.672	1	0.102
Rate of Agonistic Bouts on Land	Intercept	−1.206	2.013	−0.599	0.549	0.359	1	0.549
	Barrier Presence (Pre)	−0.654	0.509	−1.284	0.199	1.649	1	0.199
	Time of Day (Morning)	0.402	0.770	0.521	0.602	2.673	2	0.263
	Time of Day (Midday)	−0.637	0.811	−0.786	0.432	-	-	-
	Temperature	−0.014	0.028	−0.494	0.622	0.244	1	0.622
	Month (November)	−0.575	0.565	−1.017	0.309	1.034	1	0.310
Proportion of Group in Water	Intercept	1.478	0.464	3.184	0.001 *	10.140	1	0.001 *
	Barrier Presence (Pre)	0.372	0.138	2.693	0.007 *	7.251	1	0.007 *
	Time of Day (Morning)	0.928	0.126	7.337	<0.001 *	166.543	2	<0.001 *
	Time of Day (Midday)	−0.294	0.098	−3.015	0.003 *	-	-	-
	Temperature	−0.036	0.006	−5.769	<0.001 *	33.278	1	<0.001 *
	Month (November)	−0.035	0.146	−0.242	0.809	0.059	1	0.809
Proportion of Group in Proximity	Intercept	0.194	0.408	0.475	0.635	0.225	1	0.635
	Barrier Presence (Pre)	−0.409	0.110	−3.702	<0.001 *	13.707	1	<0.001 *
	Time of Day (Morning)	−0.798	0.112	−7.104	<0.001 *	57.583	2	<0.001 *
	Time of Day (Midday)	−0.198	0.087	−2.283	0.022 *	-	-	-
	Temperature	−0.008	0.006	−1.389	0.165	1.929	1	0.165
	Month (November)	0.122	0.118	1.031	0.303	1.063	1	0.303

* Denotes statistical significance (α ≤ 0.05).

## References

[1] Lawrence K., Sherwen S.L., Larsen H. (2021). Natural habitat design for zoo-housed elasmobranch and teleost fish species improves behavioural repertoire and space use in a visitor facing exhibit. Animals.

[2] Glaeser S.S., Shepherdson D., Lewis K., Prado N., Brown J.L., Lee B., Wielebnowski N. (2021). Supporting zoo Asian elephant (*Elephas maximus*) welfare and herd dynamics with a more complex and expanded habitat. Animals.

[3] Ross S.R., Calcutt S., Schapiro S.J., Hau J. (2011). Space use selectivity by chimpanzees and gorillas in an indoor-outdoor enclosure. Am. J. Primatol..

[4] Koyama N.F., Aureli F. (2019). Social network changes during space restriction in zoo chimpanzees. Primates.

[5] Ross S.R., Wagner K.E., Schaprio S.J., Hau J. (2010). Ape behavior in two alternating environments: Comparing exhibit and short-term holding areas. Am. J. Primatol..

[6] Ritzler C.P., Lukas K.E., Bernstein-Kurtycz L.M., Koester D.C. (2021). The effects of choice-based design and management on the behavior and space use of zoo-housed amur tigers (*Panthera tigris altaica*). J. Appl. Anim. Welf. Sci..

[7] Lukas K.E., Hoff M.P., Maple T.L. (2003). Gorilla behavior in response to systematic alternation between zoo enclosures. Appl. Anim. Behav. Sci..

[8] Carlstead K., Shepherdson D. (1994). Effects of environmental enrichment on reproduction. Zoo Biol..

[9] Carlstead K., Shepherdson D., Moberg G.P., Mench J.A. (2000). Alleviating stress in zoo animals with environmental enrichment. The Biology of Animal Stress: Basic Principles and Implications for Animal Welfare.

[10] Arey D.S., Edwards S.A. (1998). Factors influencing aggression between sows after mixing and the consequences for welfare and production. Livest. Prod. Sci..

[11] Deeming C., Cooper J., Hodges H. (2011). Effect of sight barriers in pens of breeding ring-necked pheasants (*Phasianus colicus*): Behavior and welfare. Br. Poult. Sci..

[12] Torrezani C.S., Pinho-Neto C.F., Miyai C.A., Sanches F.H.C., Barreto R.E. (2013). Structural enrichment reduces aggression in Tilapia rendalli. Mar. Freshw. Behav. Physiol..

[13] Valuska A.J., Leighty K.A., Shutz P.J., Ferrie G.M., Sky C.C., Bettinger T.L. (2013). The use of visual barriers to reduce aggression among a group of marabou storks (*Leptoptilos crumeniferus*). Zoo Biol..

[14] Troxell-Smith S.M., Miller L.J. (2016). Using natural history information for zoo animal management: A case study with okapi. J. Zoo Aquar. Res..

[15] Larson J.M. (2017). Mitigation of Alarm Calls and Vigilant Behavior in Captive Brown Capuchin Monkeys (*Cebus paella*): Using a Visual Barrier to Reduce Stress from a Nearby Canada Lynx (*Lynx canadensis*). Master’s Thesis.

[16] Flanagan A.M., Rutz C., Farabaugh S., Greggor A.L., Masuda B., Swaisgood R.R. (2020). Inter-aviary distance and visual access influence conservation breeding outcomes in a territorial, endangered bird. Biol. Conserv..

[17] Sherwen S.L., Hemsworth P.H. (2019). The visitor effect on zoo animals: Implications and opportunities for zoo animal welfare. Animals.

[18] Chiew S.J., Butler K.L., Sherwen S.L., Coleman G.J., Fanson K.V., Hemsorth P.H. (2019). Effects of regulating visitor viewing proximity and the intensity of visitor behaviour on the little penguin (*Eudyptula minor*) behaviour and welfare. Animals.

[19] Blaney E.C., Wells D.L. (2004). The influence of a camouflage net barrier on the behavior, welfare and public perceptions of zoo-housed gorillas. Anim. Welf..

[20] Clark F.E., Fitzpatrick M., Hartley A., King A.J., Lee T., Routh A., Walker S.L., George K. (2012). Relationship between behavior, adrenal activity, and environment in zoo-housed western lowland gorillas (*Gorilla gorilla gorilla*). Zoo Biol..

[21] Kuhar C.W., Bettinger T.L., Sironen A.L., Shaw J.H., Lasley B.L. (2003). Factors affecting reproduction in zoo-housed Geoffroys’ tamarins (*Saguinus geoffroyi*). Zoo Biol..

[22] Bashaw M.J., Kelling A.S., Bloomsmith M.A., Maple T.L. (2007). Environmental effects on the behavior of zoo-housed lions and tigers, with a case study of the effects of a visual barrier on pacing. J. Appl. Anim. Welf. Sci..

[23] Martin O., Vinyoles D., Garcia-Galea E., Maté C. (2016). Improving the welfare of a zoo-housed male drill (*Mandrillus leucophaeus poensis*) aggressive towards visitors. J. Appl. Anim. Welf. Sci..

[24] Crofoot M.C., Rubenstein D.I., Maiya A.S., Berger-Wolf T.Y. (2011). Aggression, grooming and group-level cooperation in white-faced capuchins (*Cebus capucinus*): Insights from social networks. Am. J. Primatol..

[25] Cronin K.A., Tank A., Ness T., Leahy M., Ross S.R. (2020). Sex and season predict wounds in zoo-housed Japanese macaques (*Macaca fuscata*): A multi-institutional study. Zoo Biol..

[26] Leeds A., Boyer B., Ross S.R., Lukas K.E. (2015). The effects of group type and young silverbacks on wounding rates in western lowland gorilla (*Gorilla gorilla gorilla*) groups in North American Zoos. Zoo Biol..

[27] Leeds A., Boyer B., Ross S.R., Lukas K.E. (2021). Patterns of wounding in mixed-sex social groups of western lowland gorillas (*Gorilla gorilla gorilla*). Appl. Anim. Behav. Sci..

[28] Ross S.R., Bloomsmith M.A., Bettinger T.L., Wagner K.E. (2009). The influence of captive adolescent male chimpanzees on wounding: Management and welfare implications. Zoo Biol..

[29] Wiley J.N., Leeds A., Carpenter K.D., Kendall C.J. (2018). Patterns of wounding in hamadryas baboons (*Papio hamadryas*) in North American zoos. Zoo Biol..

[30] Brien M.L., Gienger C.M., Webb G.J., McGuinness K., Christian K.A. (2014). Out of sight or in too deep: Effect of visual barriers and water depth on agonistic behavior and growth in hatchling saltwater crocodiles (*Crocodylus porosus*). Appl. Anim. Behav. Sci..

[31] Nagloo N., Collin S.P., Hemmi J.M., Hart N.S. (2016). Spatial resolving power and spectral sensitivity of the saltwater crocodile, *Crocodylus porosus*, and the freshwater crocodile, *Crocodylus johnstoni*. J. Exp. Biol..

[32] Garrick L.D., Lang J.W. (1977). Social signals and behaviors of adult alligators and crocodiles. Am. Zool..

[33] Fergusson R.A., Manolis S.C., Stevenson C. (2010). Nile Crocodile *Crocodylus niloticus*. Crocodiles: Status Survey and Conservation Action Plan.

[34] Kofron C.P. (1993). Behavior of Nile crocodiles in a seasonal river in Zimbabwe. Copeia.

[35] Modha M.L. (1967). The ecology of the Nile crocodile (*Crocodylus niloticus laurenti*) on Central Island, Lake Rudolf. E. Afr. Wildl. J..

[36] Pooley A.C., Gans C. (1976). The Nile crocodile. Sci. Am..

[37] Riley A., Terry M., Freeman H., Alba A.C., Soltis J., Leeds A. (2021). Evaluating the effect of visitor presence on Nile crocodile (*Crocodylus niloticus*) behavior. J. Zool. Bot. Gard..

[38] Wark J.D., Cronin K.A., Niemann T., Shender M.A., Horrigan A., Kao A., Ross M.R. (2019). Monitoring the behavior and habitat use of animals to enhance welfare using the ZooMonitor app. Anim. Behav. Cogn..

[39] Brien M.L., Lang J.W., Webb G.J., Stevenson C., Christian K.A. (2013). The good, the bad, and the ugly: Agonistic behaviour in juvenile crocodilians. PLoS ONE.

[40] R Core Team (2021). R: A Language and Environment for Statistical Computing.

[41] Brooks M.E., Kristensen K., van Benthem K.J., Magnusson A., Berg C.W., Nielsen A., Skaug H.J., Maechler M., Bolker B.M. (2017). glmmTMB balances speed and flexibility among packages for zero-inflated generalized linear mixed modeling. R J..

[42] Quinn G.P., Keough M.J. (2002). Experimental Design and Data Analysis for Biologists.

[43] Weldon P.J., Ferguson M.W.J. (1993). Chemoreception in crocodilians: Anatomy, natural history and empirical results. Brain Behav. Evol..

[44] Kofron C.P. (1990). The reproductive cycle of the Nile crocodile (*Crocodylus niloticus*). J. Zool..

[45] Detoeuf-Boulade A.S. (2006). Reproductive Cycle and Sexual Size Dimorphism of the Nile Crocodile (*Crocodylus niloticus*) in the Okavango Delta, Botswana. Master’s Thesis.

[46] Seebacher F., Grigg G.C., Beard L.A. (1999). Crocodiles as dinosaurs: Behavioural thermoregulation in very large ectotherms leads to high and stable body temperatures. J. Exp. Biol..

[47] Pough F.H., Aspey W.P., Lustick S. (1983). Amphibians and reptiles as low-energy systems. Behavioural Energetics: The Cost of Survival in Vertebrates.

[48] Agha M., Price S.J., Nowakowski J., Augustine B., Todd B.D. (2017). Mass mortality of eastern box turtles with upper respiratory disease following atypical cold weather. Dis. Aquat. Org..

[49] Estep D.Q., Baker S.C. (1991). The effects of temporary cover on the behaviour of socially housed stump-tailed macaques (*Macaca arctoides*). Zoo Biol..

[50] (2021). Species360 Zoological Information Management System. Zims.Species360.org.

